# Immunogenicity and Protective Efficacy of a Fusion Protein Tuberculosis Vaccine Combining Five Esx Family Proteins

**DOI:** 10.3389/fcimb.2017.00226

**Published:** 2017-05-31

**Authors:** Zhi-hao Xiang, Rui-feng Sun, Chen Lin, Fu-zeng Chen, Jun-tao Mai, Yu-xiao Liu, Zi-yan Xu, Lu Zhang, Jun Liu

**Affiliations:** ^1^State Key Laboratory of Genetic Engineering, School of Life Science, Institute of Genetics, Fudan UniversityShanghai, China; ^2^Department of Molecular Genetics, University of TorontoToronto, ON, Canada; ^3^Key Laboratory of Medical Molecular Virology of Ministries of Education and Health, Fudan UniversityShanghai, China; ^4^Shanghai Engineering Research Center of Industrial MicroorganismsShanghai, China

**Keywords:** tuberculosis, vaccine, Esx family protein, subunit vaccine, fusion protein

## Abstract

One strategy to develop the next generation of tuberculosis vaccines is to construct subunit vaccines based on T cell antigens. In this study, we have evaluated the vaccine potential of a fusion protein combining EsxB, EsxD, EsxG, EsxU, and EsxM of *Mycobacterium tuberculosis* (*M. tb*). This recombinant protein, named BM, was expressed in and purified from *Escherichia coli*. Immunization of C57BL/6 mice with purified BM protein formulated in Freund's incomplete adjuvant induced the production of Th1 cytokines (IFN-γ, TNF, and IL-2) and multifunctional CD4^+^ T cells. Vaccination of BALB/c mice with BM protein followed by intravenous challenge with *Mycobacterium bovis* BCG resulted in better levels of protection than the two leading antigens, Ag85A and PPE18. Taken together, these results indicate that BM is a protective antigen. Future studies to combine BM with other antigens and evaluate its effectiveness as a booster of BCG or as a therapeutic vaccine are warranted.

## Introduction

Tuberculosis (TB) ranks alongside HIV/AIDS as a major cause of mortality by infectious diseases worldwide. As much as one-third of the world's population is latently infected with *M. tb* and 1.5 million people died from TB in 2014. The situation has been further complicated with the advent of *M. tb*/HIV co-infection and the emergence of drug resistant TB. Bacille Calmette-Guérin (BCG), an attenuated strain of *Mycobacterium bovis*, is the only vaccine available for TB control. However, BCG exhibits highly variable efficacy against adult pulmonary TB (Brewer, 2000) and can cause disseminated BCG disease in immunocompromised individuals (WHO, 2007). In light of these situations, a more effective vaccine is urgently needed, which is essential to reduce the estimated 8–10 million new TB infections that occur annually.

Currently, it is thought that the next generation of TB vaccines will use a heterologous prime-boost strategy to strengthen the immune response introduced by BCG (Skeiky and Sadoff, 2006; Kaufmann, 2011). This “prime-boost” strategy would include administration of BCG or recombinant BCG, the “prime,” followed by a “booster” inoculation with a subunit vaccine (protein/peptide or DNA) to infants and young children before they are exposed to TB. This subunit vaccine could also be administered as a separate booster to young adults or as an adjunct to chemotherapy (Skeiky and Sadoff, 2006; Kaufmann, 2011).

A key aspect of the strategy concerns the selection of antigens to be used in constructing these subunit vaccines. Protection against TB requires a cell-mediated immune response, which is not fully understood but involves multiple components including CD4^+^ and CD8^+^ T cells, as well as unconventional T cells such as γδ T cells and CD1-restricted αβ T cells (North and Jung, 2004; Cooper, 2009; Andersen and Kaufmann, 2014). Currently, there is no proven immunological correlate of protection or “biomarker” for efficacy (Mittrucker et al., 2007; Soares et al., 2008; Nunes-Alves et al., 2014). BCG has been shown to induce a T helper cell 1 (Th1) type response, mostly IFN-γ by CD4^+^ T cells (Black et al., 2002). IFN-γ plays a critical role in the control of TB in both mice (Cooper et al., 1993; Flynn et al., 1993) and humans (Jouanguy et al., 1996; Newport et al., 1996). As such, the identification of *M. tb* antigens that induce strong IFN-γ production has been the main strategy for uncovering candidates for construction of subunit vaccines. Some antigens that have been identified thus far include the Esx family proteins (EsxA, B, G, H, G, N), the antigen 85 family (Ag85A, B, C), and several PE/PPE family proteins (e.g., PPE18, PPE14) (Borremans et al., 1989; Bassey et al., 1996; Coler et al., 1998; Brandt et al., 2000; Brodin et al., 2004; Dietrich et al., 2005). Three fusion proteins H1 (Ag85B-EsxA), H4 (Ag85B-EsxH), and M72 (PPE18-Rv0125) were constructed and have since entered clinical trials (Andersen and Kaufmann, 2014).

To stimulate greater CD8^+^ T cell recognition of antigens, DNA-based subunit vaccines have also been exploited, using replication-deficient viral vectors such as adenovirus or vaccinia virus for delivery. This is exemplified by MVA85A, a vaccinia virus expressing Ag85A; and AERAS-402, an adenovirus-35 expressing Ag85A, Ag85B, and EsxH (Andersen and Kaufmann, 2014). MVA85A has recently completed phase IIb trial, the first candidate to reach efficacy testing in clinical trials. Unfortunately, the results are disappointing (Tameris et al., 2013). In BCG-vaccinated South African infants, MVA85A did not significantly improve on BCG alone in terms of protection, either against TB disease or *M. tb* infection (Tameris et al., 2013). As such, it is necessary to continue searching for new antigens or novel combination of antigens for the pipeline. Here, we describe the immunogenicity and protective efficacy of a fusion protein that combines five EsxB-like proteins.

## Materials and methods

### Bacterial strains and culture conditions

*Mycobacterium bovis* BCG-Pasteur was grown at 37°C in Middlebrook 7H9 broth (Difco™) supplemented with 0.2% glycerol, 10% albumin-dextrose-catalase (ADC; BD BBL™), and 0.05% Tween 80 or on Middlebrook 7H11 agar (Difco™) supplemented with 0.5% glycerol and 10% oleic acid-albumin-dextrose-catalase (OADC; BD BBL™). *Escherichia coli* strain DH5α was used for routine manipulation and propagation of plasmid DNA and strain BL21 was used for expression and purification of recombinant proteins. *E. coli* strains were grown in LB broth or agar.

### Molecular cloning

The DNA fragment for expressing the BM fusion protein was constructed by linking *esxB*-*esxD*-*esxG*-*esxU*-*esxM* in tandem in the linear order. A 9-amino acid linker (GGACTAGTACCCCGAGGATCAACAGGA) was added between each two adjoining components of the polyprotein. The ORFs encoding *esxB, esxD, esxG, esxU*, and *esxM* were amplified by PCR using the primers in Table S1 and the genomic DNA of *M. tb* H37Rv as the template. These five fragments were linked together and cloned into pUC19 vector by ClonExpress MultiS One Step Cloning Kit (Vazyme). After extracting the recombinant plasmid, BM full length DNA fragment was amplified by PCR using the appropriate primers (Table S1). The fragment was then cloned into pET28a by restriction digestion and ligation. Genes encoding three other proteins, PPE18, Ag85A and EsxB, were cloned into pET28a or pET SUMO using the appropriate primers (Table S1). Recombinant plasmids were transformed into *E. coli* DH5α and plated on LB agar containing 50 μg/ml kanamycin. Single colonies were randomly picked and grown in LB broth. The plasmids were isolated from *E. coli* DH5α cultures and confirmed by DNA sequencing.

### Expression and purification of recombinant proteins

To express the recombinant protein, the pET28 or pET SUMO constructs were individually transformed into *E. coli* BL21 and plated on LB agar containing kanamycin (50 μg/ml). After overnight incubation at 37°C, single colonies were randomly picked and grown in LB broth and subcultured to 1 L. To induce the expression of protein, *E. coli* BL21 cultures were grown at 37°C to OD_600_ = 0.8 and added with 1 mM IPTG for 3 h. For Ag85A and EsxB proteins, which were purified from the soluble fraction, the cultures were collected by centrifugation at 12,000 rpm for 10 min at 4°C, and resuspended with BugBuster protein extraction reagent (Novagen). The cell lysates were then centrifuged at 12,000 rpm for 20 min at 4°C and the supernatant was collected. The collected supernatant was subjected to Ni-NTA His•Bind® Resin (Novagen) and purified following the protocol recommended by the manufacturer. The SUMO tag was digested from the purified proteins by ULP1. For BM and PPE18 proteins, which were purified from inclusion body, the cultures collected after IPTG induction were resuspended with PBS and sonicated to break the cells. The cell lysates were centrifuged at 12,000 rpm for 20 min at 4°C and the pellet was collected. The collected pellet was resuspended with 20 mM Tris, pH 8.0, 100 mM NaCl, and 8 M urea, which was then subjected to Ni-NTA His•Bind® Resin. Purified proteins were centrifuged using microfuge tubes with a low molecular weight cut off filter to remove urea and imidazole. The protein purity was verified by polyacrylamide gel electrophoresis, and the protein concentration was measured by the Bradford assay (Bio-Rad).

### Immunogenicity assays

All of the animal procedures were approved by the local animal care committees at Fudan University. To determine the induction of Th1 cytokines, C57BL/6 mice (4 per group) were immunized subcutaneously with a mixture containing 100 μl each of the purified protein (10 μg) and 100 μl of Freund's incomplete adjuvant (Sigma). The vaccination procedure was repeated two more times (2 weeks apart). At week 8 after the first vaccination, the mice were sacrificed and their spleens were collected. Splenocytes were prepared in 24-well plate, with each well containing 10^6^ lymphocytes. The corresponding purified proteins were added to the wells at two different concentrations (5 and 10 μg/ml). The plates were incubated at 37°C with 5% CO_2_ for 60 h. The cell supernatants were harvested and the production of Th1 cytokines (IFN-γ, TNF, IL-2) was determined by ELISA using the OptEIATM ELISA Kit (BD Biosciences) with appropriate mAbs, according to the protocol recommended by the manufacturer.

To determine the induction of multifunctional T cells, C57BL/6 mice (4 per group) were immunized as the above. Lymphocytes (10^6^ cells per well) were stimulated with the corresponding purified proteins (5 μg/mL) and incubated at 37°C 5% CO_2_ for 12 h. Brefeldin A was used to block the Golgi apparatus 4 h before staining. Fluorescent monoclonal antibodies (1 μL) for CD3 and CD4 (BD Biosciences) were added to each wells for the staining of surface markers. The cells were then permeabilized and fixed with CytoFix/CytoPerm (BD Biosciences). Fluorescent antibodies (1 μL) for IFN-γ, TNF and IL-2 (BD Biosciences) were added for intracellular staining. The cells were then analyzed on a Gallios flow cytometer (Beckman Coulter).

### Animal protection assays

BALB/c mice (5 per group) were immunized as described above in the “immunogenicity assays.” Eight weeks after the first vaccination, the mice were infected intravenously via a lateral tail with 10^7^ CFU (colony forming unit) of *Mycobacterium bovis* BCG-Pasteur. After 3 weeks of infection, the mice were sacrificed and their lungs and spleens were collected. The CFUs of BCG in the lungs and spleen were determined by serial dilution and platting on 7H11 agar plates (OADC).

## Results

### Generation of BM5 fusion protein

**Figure 1:**
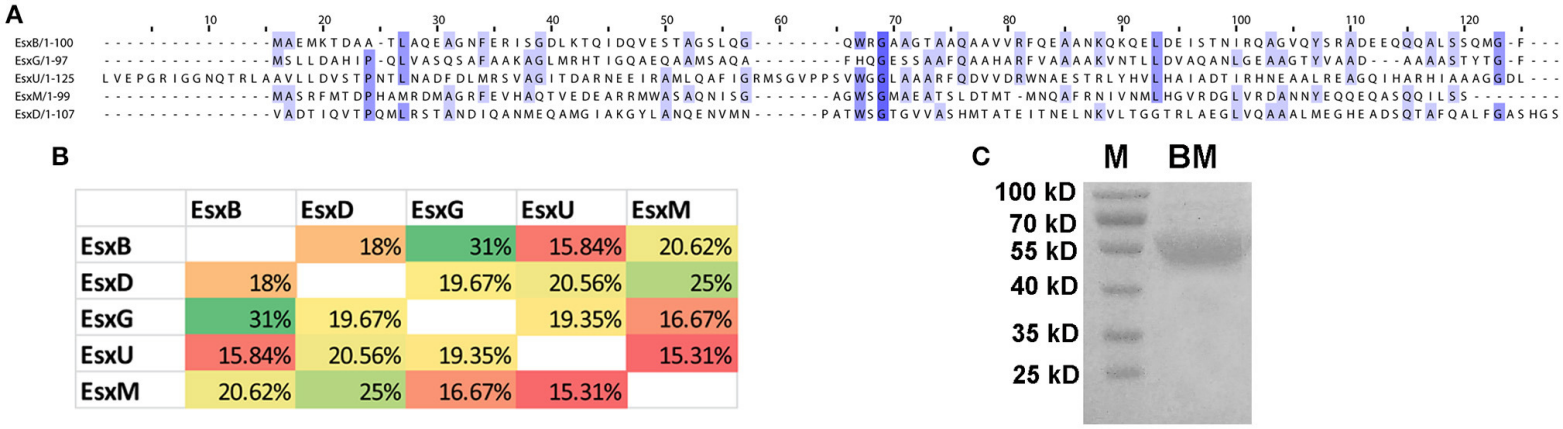
Sequence alignment of five EsxB-like proteins. **(A)** ClustalWS alignment of EsxB, EsxD, EsxG, EsxU, and EsxM. **(B)** Pairwise comparison of the sequence identity of the five EsxB-like proteins. **(C)** Purified recombinant BM fusion protein. M, molecular weight marker.

The *M. tb* genome contains 23 *esx* genes (*esxA*-*W*), which are arranged in tandem pairs in 11 genomic loci, including five ESX loci flanked by components of type VII secretion (T7S) systems (ESX-1 to -5) (Cole et al., 1998; Abdallah et al., 2007; Bitter et al., 2009). The Esx family proteins are small (~100 amino acids) secreted proteins that are substrates of the T7S systems. The first Esx protein that was discovered was the EsxA (ESAT-6) protein (Sorensen et al., 1995). EsxA and its protein partner EsxB (CFP-10) form a heterodimer, which is exported by the ESX-1 system encoded by genes flanking *esxA-esxB* (Renshaw et al., 2002; Abdallah et al., 2007). Similar genetic organizations were found in the other four ESX loci (Abdallah et al., 2007). Many of the Esx family proteins are immunodominant T cell antigens (Skjot et al., 2000; Pallen, 2002), and three of them (EsxA, EsxB, and EsxH) have been used to construct subunit vaccines that have entered clinical trials (Andersen and Kaufmann, 2014). The Esx family proteins are also called the WxG100 proteins, based on a short conserved motif in the middle of the protein, tryptophan-X-glycine (WxG), which forms the turn that connects two helices (Pallen, 2002; Renshaw et al., 2005; Poulsen et al., 2014). Interestingly, despite their similarity in genetic organization and structural fold, the Esx family proteins have low sequence homology. For example, the EsxB-like proteins in the five ESX loci (EsxB, D, G, U, and M) (Bitter et al., 2009) share <32% sequence identity by pairwise comparisons (Figures 1A,B). Five other EsxB-like proteins elsewhere in the genome do share high levels of sequence identity to their homologs in the five ESX loci. EsxJ, K, P, and W proteins are nearly identical to EsxM in the ESX-5 locus, and EsxS is highly homologous to EsxG in the ESX-3 locus. A similar result was found if we compare the EsxA-like proteins in the five ESX loci and elsewhere in the genome.

We hypothesized that a fusion protein combing Esx proteins with diverse sequences would have more T cell epitopes and would be more immunogenic and protective. To test this, we generated a fusion protein combining five EsxB-like proteins in the ESX loci, namely, EsxB, D, G, U, and M. These genes were cloned in tandem with a linker sequence between them, and the DNA fragment was inserted into pET28a and expressed in *E. coli*. A recombinant protein of ~55 KD was purified by affinity chromatography, which is in agreement with its predicted size (Figure 1C).

### BM induces the production of Th1 cytokines

To assess immunogenicity, C57BL/6 mice (4 per group) were immunized three times (2 weeks apart) with 10 μg purified BM protein formulated with Freund's incomplete adjuvant. Four weeks after the last vaccination, the mice were sacrificed and their spleens were collected. Splenocytes were prepared and cultured with or without the corresponding protein at two different concentrations (5 and 10 μg/ml). Three days after antigen stimulation, the cell supernatants were harvested and the production of Th1 cytokines (IFN-γ, TNF, IL-2) were determined by ELISA. Purified Ag85A was also included in the parallel experiment for comparison and as a positive control.

**Figure 2:**
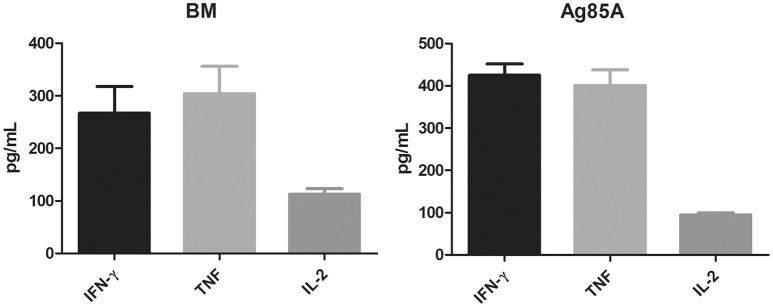
BM induces the secretion of Th1 cytokines. C57BL/6 mice were immunized three times (2 weeks apart) with 10 μg purified BM or Ag85A protein formulated with Freund's incomplete adjuvant. At 8 weeks post-vaccination, splenocytes were harvested and incubated with purified BM or Ag85A at two different concentrations (5 and 10 μg/ml) for 60 h and the productions of Th1 cytokines was analyzed by ELISA. Data were plotted as mean ± SEM (*n* = 4 mice).

Results showed that BM was highly immunogenic. It induced significant levels of Th1 cytokines including IFN-γ, TNF, and IL-2, which were comparable to the levels of these cytokines induced by Ag85A (Figure 2). Both proteins induced equally high levels of IFN-γ and TNF but lower levels of IL-2.

### BM induces multifunctional CD4^+^ T cells

Recent reports from a number of different disease models have shown that multifunctional T cells are superior to their single-positive counterparts (Seder et al., 2008). In mice, vaccine-induced multifunctional CD4^+^ T cells have been shown to correlate with protection against *Leishmania major* infection (Darrah et al., 2007). Inductions of multifunctional CD4^+^ T cells were also reported for TB subunit vaccines MVA85A and H1 (Darrah et al., 2007; Lindenstrom et al., 2009).

**Figure 3:**
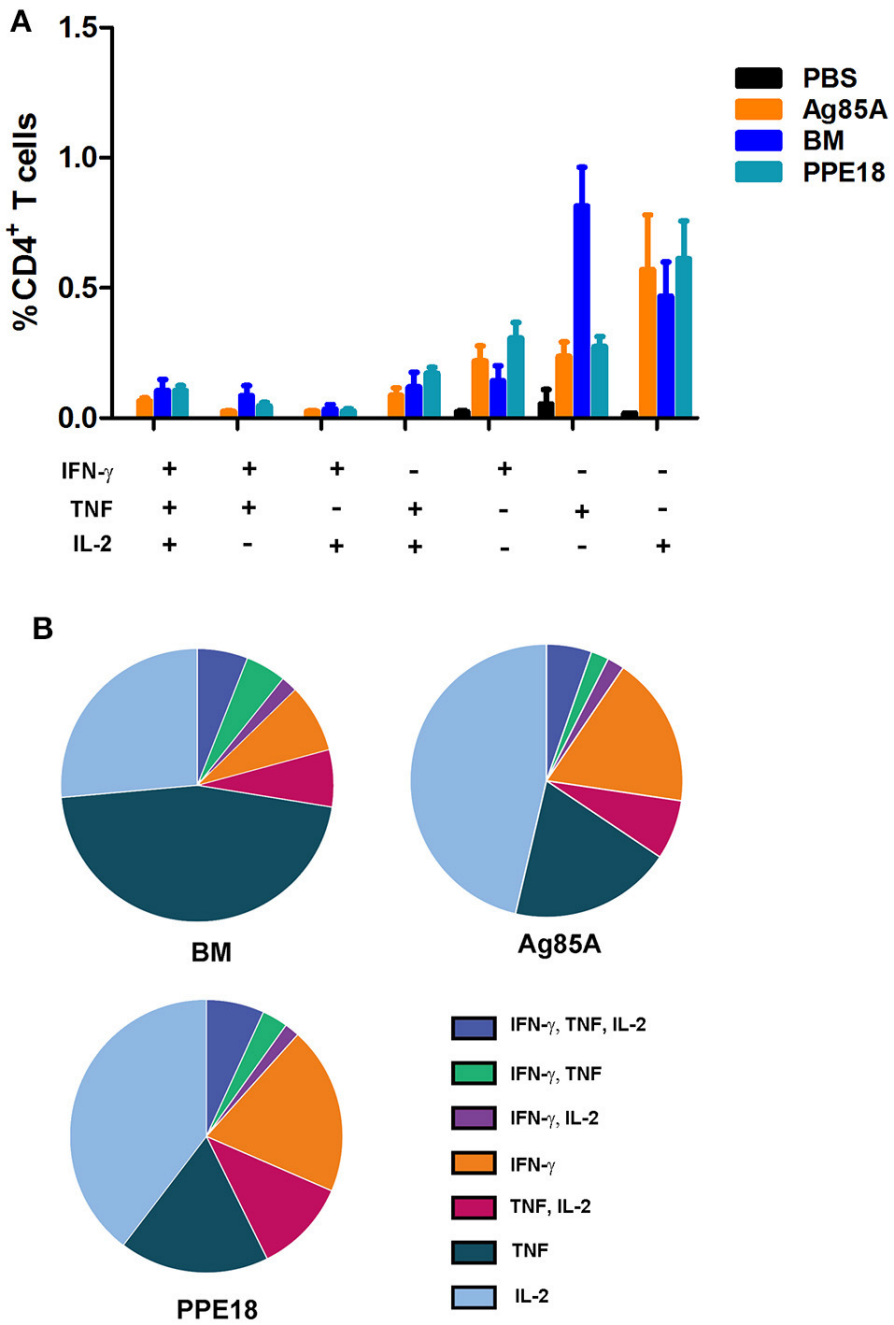
BM induces multiple functional CD4^+^ T cells. **(A)** C57BL/6 mice were immunized three times (2 weeks apart) with the indicated proteins (10 μg) formulated with Freund's incomplete adjuvant for 8 weeks. Splenocytes were isolated and stimulated with the corresponding purified protein (5 μg/ml) for 12 h and were then stained for surface markers and intracellular cytokines, followed by FACS analysis. Data were plotted as mean ± SEM (*n* = 4 mice). **(B)** Pie chart of the data.

To determine if BM induces multifunctional T cells, we repeated the immunization of CL57/BL6 mice with BM and then measured the production of CD4^+^ T cells expressing multiple effectors including IFN-γ, TNF, and IL-2. Ag85A and PPE18 were included in this experiment for comparison. Consistent with the above result, BM induced CD4^+^ T cells with the concomitant production of IFN-γ, TNF, and IL-2, and the level of induction was comparable to that of Ag85A and PPE18 (Figure 3).

### Protective efficacy of BM fusion protein

**Figure 4:**
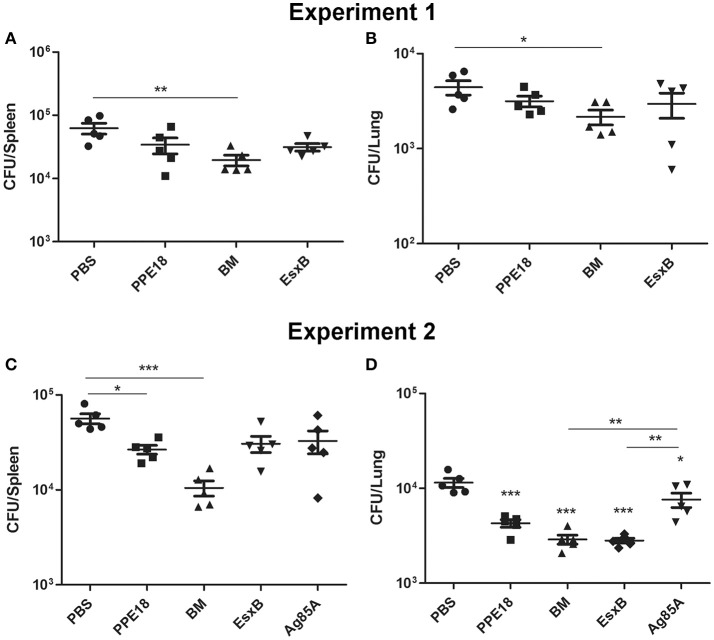
Protective efficacy of BM in intravenous infection model. BALB/c mice (5 per group) were immunized with the indicated proteins (10 μg) formulated with Freund's incomplete adjuvant for 8 weeks. Mice were then intravenously challenged with 10^7^ CFUs of *M. bovis* BCG-Pasteur. At week 3 after injection, mice were sacrificed and their lungs and spleens were collected and homogenization for BCG colony counting. Data from two independent experiments were presented. Data were plotted as mean ± SEM (*n* = 5 mice). In **(A,B)** One-Way ANOVA and Bonferroni comparison tests were performed to compare the BM and PBS groups in each dataset. ^*^p <0.05, ^**^p <0.01. In **(C,D)** One-Way ANOVA and Bonferroni comparison tests were performed to compare all groups. In **(D)** the groups of BM, EsxB, and PPE were significantly different to the PBS group (^***^p <0.001). The Ag85A group was also significantly different to the PBS group (^*^p <0.05).

To determine the protective efficacy of BM, BALB/c mice (5 per group) were immunized subcutaneously three times (2 weeks apart) with purified BM, PPE18, or EsxB formulated with Freund's incomplete adjuvant for 8 weeks. Mice were then intravenously challenged with 10^7^ CFUs of *M. bovis* BCG-Pasteur. Three weeks after injection, the mice were sacrificed and bacterial burdens in the lungs and spleen were determined. Results showed that mice immunized with BM had significantly lower BCG counts in the spleen than the unvaccinated PBS control (*p* <0.01, One-Way ANOVA) (Figure 4A). The mean BCG burden in the BM group (4.29 log_10_ CFU) was ~0.5 log_10_ lower than in the PBS control group (4.79 log_10_ CFU). Lower BCG burden (3.33 log_10_) was also found in the lungs of mice immunized with BM, compared to the PBS group (3.65 log_10_ CFU), and the difference was statistically significant (Figure 4B).

We repeated this experiment and also included Ag85A for comparison. Consistently, among all the vaccine candidates tested, the BM group had the lowest bacterial burden in the spleen. The mean BCG burden in the spleen of the BM group was 4.02 log_10_, which was 0.73 log_10_, 0.49 log_10_, 0.46 log_10_, and 0.40 log_10_ lower than the PBS, Ag85A, EsxB, and PPE groups, respectively. The difference between the BM group and the PBS group was statistically different (Figure 4C). The bacterial burden in the lungs of the BM group was also significantly lower than the PBS (ΔCFU = 0.60 log_10_, *p* <0.0001) and Ag85A groups (ΔCFU = 0.42 log_10_, *p* <0.01) (Figure 4D).

## Discussion

TB vaccine research has gained momentum in the past decade, with a dozen candidates currently under clinical trial evaluations (Andersen and Kaufmann, 2014). However, there is no guarantee that the leading candidates will progress through phase III clinical trials and registration. As previously mentioned, MVA85A, the first vaccine candidate that reached efficacy testing in clinical trials, failed to demonstrate improved protection in BCG-immunized infants (Tameris et al., 2013). Several explanations have been proposed (Beverley, 2013; Andersen and Kaufmann, 2014), including the modest magnitude of immune response induced by MVA85A in children (Tameris et al., 2013) and that the Ag85 family antigens were downregulated to very low levels soon after *M. tb* infection and not preferentially recognized by the immune system (Aagaard et al., 2011; Commandeur et al., 2013). Given the failure of MVA85A, recent efforts have focused on identifying more effective antigens and novel combination of antigens. Several studies suggest that subunit vaccines that combine multistage antigens may be a better strategy to develop effective vaccines (Bertholet et al., 2010; Aagaard et al., 2011). For example, the fusion protein H56, which combines a latency associated antigen Rv2660c with Ag85B and EsxA, conferred better protection than H1 (Ag85B-EsxA) in mice at late-stage of *M. tb* infection (Aagaard et al., 2011).

In this study, we took advantage of the sequence diversity among some of the Esx proteins and their relative small size, and constructed a fusion protein that combines five different Esx proteins. The Esx family proteins are immunodominant T cell antigens (Skjot et al., 2000; Pallen, 2002) and therefore good candidates for constructing subunit vaccines. The diverging sequence among Esx proteins in the five ESX loci is consistent with the diverse function of the T7S systems. ESX-1 is required for mycobacterial virulence (Abdallah et al., 2007), ESX-3 plays a role in iron and zinc uptake (Serafini et al., 2009; Siegrist et al., 2014), and ESX-5 is involved in the export of PE/PPE proteins and pathogenicity (Abdallah et al., 2009). The functions of ESX-2 and ESX-4 remain unknown. ESX-4, which harbors a smaller number of genes than other ESX loci, appears to be the most ancestral T7S system in mycobacteria (Gey Van Pittius et al., 2001). Other ESX loci may have evolved later by gene duplication and diversification events, which results in sequence variation and function diversity.

Our results demonstrated that BM induces potent Th1 cytokines and CD4^+^ T cells, and confers protection against mycobacterial infection. Compared with Ag85A, PPE18, and EsxB, which have been used in subunit vaccines that are in clinical trial evaluation, BM exhibits better protection. The enhanced protection of BM could be attributed to more T cell epitopes in this fusion protein than other antigens of similar size. Our results suggest that BM is an effective antigen that could be included in the construction of novel TB vaccines. BM could be combined with latency associated antigens to construct multistage subunit vaccines, or to be expressed in BCG for the construction of live vaccines. Future studies to evaluate the effectiveness of BM as a booster of BCG or as a post-exposure vaccine will determine its full potential.

## Author contributions

JL conceived and designed the project. LZ supervised the experiments. ZhX, RS, CL, FC, JM, YL, ZyX performed the experiments. JL wrote the manuscript.

### Conflict of interest statement

The authors declare that the research was conducted in the absence of any commercial or financial relationships that could be construed as a potential conflict of interest.
