# Supplementary material for: Immunogenicity and Protective Efficacy of a Fusion Protein Tuberculosis Vaccine Combining Five Esx Family Proteins
Source: Front Cell Infect Microbiol. 2017 May 31;7:226. doi: 10.3389/fcimb.2017.00226 (PMC5449442; doi:10.3389/fcimb.2017.00226)
Supplement: Supplementary file 1 [file Table1.PDF]

Table S1. Primers for cloning.

Gene	vector	Primer	Primer sequence (5'-3')
esxB	pUC19	forward	CCAGTGAATTCATGGCAGAGATGAAGACCGATGCCG
		reverse	GATCCTCGGGGTACTAGTCCGAAGCCCATTTGCGAGGACAGCG
esxD	pUC19	forward	GGACTAGTACCCCGAGGATCAACAGGAGTGGCAGACACAATTCAGGTAACACCGC
		reverse	GATCCTCGGGGTACTAGTCCGGATCCGTGGCTAGCGCCGA
esxG	pUC19	forward	GGACTAGTACCCCGAGGATCAACAGGAATGAGCCTTTTGGATGCT
		reverse	TCCGAGCTCGAACCCGGTATAGGTCGA
esxU	pUC19	forward	TTCGAGCTCGGACTAGTACCCCGAGGATCAACAGGAGTGAGCACACCGAACACG
		reverse	GGGTACTAGTCCTAGGTCGCCGCCGG
esxM	pUC19	forward	GCGGCGACCTAGGACTAGTACCCCGAGGATCAACAGGAATGGCCTCACGTTTTATGACGGATCC
		reverse	GCCAAGCTTCTAGCTGCTCAGGATCTGCTGGGAG
BM	pET28a	forward	CACTGAATTCATGGCAGAGATGAAGACCGAT
		reverse	GTACAAGCTTCTAGCTGCTCAGGATCTGCTG
EsxB	pET28a	forward	ATATGGATCCATGGCAGAGATGAAGACCGATGC
		reverse	GCACAAGCTTTCAGAAGCCCATTTGCGAGGA
Ag85A	pET SUMO	forward	CGAATTCATGGCATTTTCCCGGCCGGGCTTG
		reverse	ATGAGCTCCTAGGCGCCCTGGGGCGCGGG
PPE18	pET28a	forward	GCTGGATCCATGGTGGATTTCGGGGCGTTACCAC
		reverse	TATAAGCTTCTAGCCGGCCGCCGGAGAATGCG
